# The IL-6/STAT3 Signaling Pathway Is an Early Target of Manuka Honey-Induced Suppression of Human Breast Cancer Cells

**DOI:** 10.3389/fonc.2017.00167

**Published:** 2017-08-14

**Authors:** Priyanka Aryappalli, Sarah S. Al-Qubaisi, Samir Attoub, Junu A. George, Kholoud Arafat, Khalil B. Ramadi, Yassir A. Mohamed, Mezoon M. Al-Dhaheri, Ashraf Al-Sbiei, Maria J. Fernandez-Cabezudo, Basel K. al-Ramadi

**Affiliations:** ^1^Department of Medical Microbiology and Immunology, College of Medicine and Health Sciences, United Arab Emirates University, Al Ain, United Arab Emirates; ^2^Department of Pharmacology and Therapeutics, College of Medicine and Health Sciences, United Arab Emirates University, Al Ain, United Arab Emirates; ^3^Department of Biochemistry, College of Medicine and Health Sciences, United Arab Emirates University, Al Ain, United Arab Emirates

**Keywords:** triple-negative breast cancer, manuka honey, STAT3, interleukin-6, apoptosis

## Abstract

There is renewed interest in the potential use of natural compounds in cancer therapy. Previously, we demonstrated the anti-tumor properties of manuka honey (MH) against several cancers. However, the underlying mechanism and molecular targets of this activity remain unknown. For this study, the early targets of MH and its modulatory effects on proliferation, invasiveness, and angiogenic potential were investigated using two human breast cancer cell lines, the triple-negative MDA-MB-231 cells and estrogen receptor-positive MCF-7 cells, and the non-neoplastic breast epithelial MCF-10A cell line. Exposure to MH at concentrations of 0.3–1.25% (w/v) induced a dose-dependent inhibition of the proliferation of MDA-MB-231 and MCF-7, but not MCF-10A, cells. This inhibition was independent of the sugar content of MH as a solution containing equivalent concentrations of its three major sugars failed to inhibit cell proliferation. At higher concentrations (>2.5%), MH was found to be generally deleterious to the growth of all three cell lines. MH induced apoptosis of MDA-MB-231 cells through activation of caspases 8, 9, 6, and 3/7 and this correlated with a loss of Bcl-2 and increased Bax protein expression in MH-treated cells. Incubation with MH induced a time-dependent translocation of cytochrome *c* from mitochondria to the cytosol and Bax translocation from the cytosol into the mitochondria. MH also induced apoptosis of MCF-7 cells *via* the activation of caspases 9 and 6. Low concentrations of MH (0.03–1.25% w/v) induced a rapid reduction in tyrosine-phosphorylated STAT3 (pY-STAT3) in MDA-MB-231 and MCF-7 cells. Maximum inhibition of pY-STAT3 was observed at 1 h with a loss of >80% and coincided with decreased interleukin-6 (IL-6) production. Moreover, MH inhibited the migration and invasion of MDA-MB-231 cells as well as the angiogenic capacity of human umbilical vein endothelial cells. Our findings identify multiple functional pathways affected by MH in human breast cancer and highlight the IL-6/STAT3 signaling pathway as one of the earliest potential targets in this process.

## Introduction

Breast cancer is the most prevalent cancer among women worldwide with a mortality rate of >500,000 annually, 62% of deaths occurring in developing countries (1). Despite improved screening and early detection, a favorable treatment outcome is still a challenge, particularly for triple-negative breast cancers (TNBCs). TNBCs are so named because they lack expression of estrogen receptor (ER), progesterone receptor, and human epidermal growth factor receptor-2. Patients with TNBCs have poor prognosis due to inherent resistance of their cancers to chemotherapy treatment, leading to increased risk of recurrence (2). Another major challenge in breast cancer treatment is the development of metastasis, since metastatic breast cancer cells are frequently resistant to almost all available therapies (3). Given the limitations in currently used treatment modalities, including chemotherapy, radiotherapy, and surgery, and their associated toxicities, for breast and other types of cancers, complementary/alternative medicine approaches have received increasing attention over the past few years (4–6).

Previously, we demonstrated that low concentrations of manuka honey (MH) can effectively inhibit the growth of several types of cancer cells, including melanoma, breast adenocarcinoma, and colorectal cancer (7). Moreover, using a preclinical model of implantable melanoma, systemic administration of MH enhanced the anti-tumor activity of paclitaxel and improved overall host survival (7). Several groups have also reported on the anti-tumor activity of various types of honey on cancer cells [see Ref. (8) for a recent review]. However, with few exceptions (9–11), most of these studies were carried out using *in vitro* systems. The anti-proliferative and pro-apoptotic properties of honey on cancer cells are thought to be mainly due to its phenolic compound constituents, including chrysin, luteolin, quercetin, and caffeic acid esters (12–15). We and others demonstrated that honey induces caspase-mediated apoptosis in different cancer cell lines, such as melanoma, breast, cervical, prostate, renal, and liver cancers (16–21). However, what remains largely unknown is the nature of the earliest upstream target in cancer cells that is affected by honey treatment.

For this study, we selected two human breast cancer cell lines, the triple-negative MDA-MB-231 and the ER-positive MCF-7 cells, to investigate susceptibility to MH and to identify the earliest signaling pathways affected. We demonstrate that MH prevents the growth of cancer cells in a time and dose-dependent manner. Moreover, treatment with low concentrations of MH (≤1%) led to an inhibition of cancer cell migration and invasion capacity. With regard to the potential signaling pathway involved, our study demonstrate that treatment of MDA-MB-231 and MCF-7 cancer cells with MH led to a dose- and time-dependent inhibition of pY-STAT3, which was observed as early as 15 min after cell exposure to <1% solution of MH. Importantly, treatment with MH also led to decreased interleukin-6 (IL-6) production by both cancer cell lines. These findings identify the IL-6/STAT3 signaling pathway as an early molecular target of MH in human cancer and reveal the important consequences of this inhibition on multiple effector functions of breast cancer cells.

## Materials and Methods

### Cell Lines and Reagents

Human breast cancer cell lines MDA-MB-231, MDA-MB-435, and MCF-7 were generously provided by Dr. Salem Chouaib (Institut Gustave Roussy, Villejuif, France) and were maintained in complete DMEM supplemented with 10% FCS (Hyclone-GE Healthcare life Sciences, Pittsburg, PA, USA), as previously described (7). The MCF-10A breast epithelial cell line (22) was the generous gift of Dr. Joan Brugge (Harvard Medical School, Boston, MA, USA) provided through the laboratory of Dr. Raif Geha (Boston Children’s Hospital, Boston, MA, USA). MCF-10A cells were maintained in DMEM-F12 medium supplemented with 5% horse serum (Invitrogen), 20 ng/ml EGF (Peprotech), 0.5 µg/ml hydrocortisone, 100 ng/ml cholera toxin, 10 µg/ml insulin (Sigma) (St. Louis, MO, USA) and penicillin–streptomycin (Hyclone). Paclitaxel (hereafter referred to as taxol) was purchased from Sigma and MH (UMF^®^ 16+) from ApiHealth (Auckland, New Zealand). As a control for MH, we used a sugar solution (designated sugar control or SC) containing equivalent concentrations of the three major sugars in honey (38.2% fructose, 31.3% glucose, and 1.3% sucrose) (23). For all reagents, appropriate dilutions to the desired concentrations were made fresh in culture medium before addition to the cells in culture.

### Cell Proliferation Assay

Cell viability was determined as previously detailed (7) using CellTiter-Glo^®^ Luminescent Cell Viability Assay (Promega, Madison, WI, USA). This assay quantifies the amount of ATP present as a correlate of the number of metabolically viable cells in culture. Briefly, MDA-MB-231 tumor cells (5 × 10^3^ cells/well) or MCF-10A cells (2 × 10^4^ cells/well) were cultured in 96-well plate and exposed to different concentrations of MH (range 0.25–2%; w/v) for different time periods (range 24–72 h), as indicated. At the end of culture, a cell lysis solution, containing a luciferin derivative, Ultra-Glo™ Recombinant Luciferase and Mg^2+^, was added and this converts the luciferin derivative into a luminescent signal proportional to the amount of ATP present. Luminescence was measured using a Glomax Luminometer (Promega) and normalized to control. The data are presented as percent cell viability of experimental groups compared to that of control, untreated cells.

### Caspase Assays

The activity of caspase-3/7, caspase-6, caspase-8, and caspase-9 were assayed using specific Caspase-Glo^®^ assay kits (Promega), essentially following manufacturer’s instructions (7). Briefly, MDA-MB-231 or MCF-7 cells were seeded in a 96-well U bottom plate (5 × 10^3^ cells/well) and treated with MH (1–5% w/v, final concentrations) or Taxol (50 ng/ml) for 24 h. Subsequently, cell lysis solution, containing a luciferase substrate derivative, Ultra-Glo™ Recombinant Luciferase and Mg^2+^, was added for 2 h at room temperature. For all assays, duplicate plates were set up to quantify cell viability. Luminescence was measured using a Glomax Luminometer. The luminescent signal was normalized to control, as per manufacturer’s protocol, and adjusted relative to the extent of cell viability in each group. All determinations were done in duplicate for each experimental group. The data are reported as the mean of relative fold increase compared to untreated cells.

### Western Blot Analysis

Detailed procedures for immunoblotting have been published previously (24, 25). MDA-MB-231 cells (4 × 10^6^ cells/well) were seeded overnight in 20 × 100 mm culture dish in DMEM containing 2% FBS. Cells were then gently washed and incubated in DMEM/5% FBS in the presence of MH (0.03–1% final concentration) for different times (range 15 min–12 h). At the end of incubation, cells were lysed using RIPA buffer (25 mM Tris-Cl, 150 mM sodium chloride, 1% NP-40, 0.5% sodium deoxycholate, 0.1% SDS, and protease inhibitors) and lysates clarified by centrifugation at 10,000 rpm and stored at −80°C until use. Aliquots (60–80 µg) of total proteins were resolved on 10–12% SDS-PAGE, transferred to a nitrocellulose membrane and blocked with 5% non-fat milk for 1 h. Membranes were then probed with appropriate primary antibodies (1/1,000 dilution) overnight at 4°C. The antibodies used were specific to STAT3 [Cell Signaling Technology (CST), MA, USA], pY-STAT3 (Tyr705) (CST), cytochrome *c* (CST), Bcl-2 (Santa Cruz Biotechnology, CA, USA), Bax (Santa Cruz), phosphotyrosine (mAb clone 4G10) (Upstate Biotechnology, NY, USA), VDAC (CST) or β-actin (CST). Blots were then washed and exposed to appropriate anti-mouse or anti-rabbit HRP-conjugated secondary antibodies (1:2,000; CST) and developed using the ECL Plus substrate system (Pierce-Thermo Fisher Scientific, MA, USA). The chemiluminescent band signal was detected by Typhoon FLA 9500 biomolecular imager (GE Healthcare Life Sciences). Densitometric analysis of band intensity on blots was done using ImageJ (National Institutes of Health, USA).

### Mitochondrial Lysates

Lysates of cytoplasmic and mitochondrial fractions were prepared using the Qproteome Mitochondria Isolation Kit (Qiagen), according to the manufacturer’s protocol. Briefly, MDA-MB-231 cells (5 × 10^6^ cells/well) were seeded overnight in 20 × 100 mm culture dish in DMEM containing 2% FBS. Cells were then gently washed and incubated in DMEM/5% FBS in the presence of MH or SC (1% final concentration) for 24, 48, and 72 h. After treatment, cell were harvested, washed in 0.9% NaCl, and incubated for 10 min at 4°C in lysis buffer on a shaker. The homogenate was centrifuged at 1,000 × *g* for 10 min at 4°C; this supernatant was designated as cytosolic fraction. The pellet was resuspended in disruption buffer, passed through a 26-G needle 15 times. The lysate was centrifuged at 1,000 × *g* for 10 min at 4°C and carefully transferred supernatant to a clean 1.5 ml tube. To obtain the enriched mitochondrial fraction, the supernatant was centrifuged at 6,000 × *g* for 20 min at 4°C. All buffers were supplemented with protease inhibitors at 1:100, provided within the kit. The isolated mitochondria were lysed using RIPA buffer (25 mM Tris-Cl, 150 mM sodium chloride, 1% NP-40, 0.5% sodium deoxycholate, 0.1% SDS, and protease inhibitors) and lysates clarified by centrifugation at 16,000 rpm and stored at −80°C until use.

### Analysis of IL-6 Synthesis and Secretion

MDA-MB-231 cells (4 × 10^6^ cells/well) were seeded overnight in 2% FBS DMEM growth medium. Cells were then gently washed and incubated in DMEM/5% FBS in the presence of 1 or 5% MH for 4.5 h. Brefeldin A (CST) was added at 1 mg/ml concentration for the final 4 h of incubation to block protein egress from the endoplasmic reticulum. Total cell extracts were then prepared, as described above, resolved on 10% SDS-PAGE and blots were probed with anti-IL-6 rabbit mAb (clone D3K2N; CST). The effect of MH treatment on IL-6 secretion was also analyzed. MDA-MB-231 or MCF-7 cells were seeded overnight in 2% FBS DMEM in 6-well plates (2 × 10^6^/well), and then incubated in 5% FBS medium in the presence of indicated concentrations of MH for different times (2–12 h). Cell-free culture supernatants were then collected and analyzed for IL-6 content by a specific ELISA (Biolegend, CA, USA).

### Measurement of Intracellular Reactive Oxygen Species (ROS)

Intracellular production of ROS was measured by flow cytometry using 2′,7′-dichlorofluorescein diacetate (DCF-DA) as a fluorescent probe. Cells (5 × 10^5^ cells/well) were treated with the indicated concentrations of MH (0.3–5%) or hydrogen peroxide (as a positive control) for 24 h, then washed with PBS and stained with 5 µM (DCF-DA; Sigma, MO, USA) for 30 min at 37°C in the dark. DCF-DA is a fluorogenic dye that measures ROS within intact cells. The cells were then washed, resuspended in PBS, and analyzed on a FACSCanto II (BD Biosciences, San Jose, CA, USA). The mean fluorescence intensity was quantified using CellQuest software (BD Biosciences).

### Soft-Agar Colony Growth Assay

The detailed procedure for this assay has been published (26). Briefly, a layer of Noble agar (2.4%; 1 ml) was poured into wells of a 6-well plate and allowed to set. A second layer (2.9 ml) containing 0.3% low melting Noble agar dissolved in growth media containing cells (5 × 10^3^ cells/ml) was placed on top of the first layer and allowed to set at 4°C for 5 min followed by incubation at 37°C for 30–60 min. Cells were incubated for 2 weeks to form colonies. Growth medium (2 ml) containing MH at different concentrations was then layered on top of the second layer and cells were incubated in a humidified incubator at 37°C for 1 week. At the end of the experiment, colonies were stained with 2% Giemsa stain, photographed and counted. All colonies were counted based on their size with the number of large colonies (>200 μm in diameter) being used for calculating the effect of MH on colony formation.

### Wound Healing Motility Assay

This was carried out following a previously described protocol (27). Cells were grown in 6-well plate until confluence, incubated for 10 min with Moscona buffer following which a scrape was made through the confluent monolayer with a plastic pipette tip of 1 mm diameter. The tissue culture plates were then washed and incubated at 37°C in supplemented growth medium in the presence or absence of MH and/or drug. At the bottom side of each dish, two arbitrary places were marked where the width of the wound was measured with an inverted microscope (objective ×4) (Olympus 1X71, Japan). Motility is expressed as the average ± SEM of the difference between the measurements at time 0 and subsequent determinations after 6 h.

### Matrigel Invasion Assay

The extent of invasiveness of cancer cells was determined using a Matrigel Invasion Chamber (8-µm pore size; BD Biosciences), as described previously (27). Briefly, cells (1 × 10^5^ cells in 0.5 ml of media plus treatment) were seeded into the upper chamber of the Matrigel system. The bottom chamber in the system was filled with RPMI supplemented with 10% FBS as a chemo-attractant and then incubated at 37°C for 24 h. Cells that have migrated through the Matrigel were fixed with 4% formaldehyde, stained with DAPI and counted in 25 random fields under a microscope.

### Vascular Tube Formation Assay

The effect of the various treatments on angiogenic activity was assessed using an *in vitro* assay whereby the formation of capillary-like structures was measured using human umbilical vein endothelial cells (HUVECs) plated on Matrigel-coated plates, as previously described (27). The Matrigel matrix was thawed, gently mixed to homogeneity using cooled pipettes, and diluted v/v with the EndoGRO™-MV-VEGF Complete Media Kit medium (Millipore, Temecula, CA, USA). Matrigel, supplemented with angiogenic peptides and other effectors, was then used to coat the wells of a 96-well plate (50 µl/well) and allowed to solidify prior to treatment. HUVECs (4 × 10^4^ cells/well) were added and incubated for 8 h at 37°C in 0.1 ml of EndoGRO™-MV-VEGF Complete Media Kit medium (Millipore, Temecula, CA, USA) in the presence or absence of different concentrations of MH. The cells were photographed using an inverted phase contrast photomicroscope. The tubular network growth area was compared in control and MH-treated cells. Tube formation was quantified by determining the length of tube-like structures formed in each well.

### Statistical Analysis

Statistical significance between control and treated groups was analyzed using the unpaired, two-tailed Student’s *t*-test, using the statistical program of GraphPad Prism version 6 software (San Diego, CA, USA). For multiple comparisons, we used one-way ANOVA with *post hoc* Tukey’s test (GraphPad Prism). Differences between experimental groups were considered significant when *p*-values were <0.05.

## Results

### MH Reduces the Viability of MDA-MB-231 and MCF-7 Breast Cancer Cells

**Figure 1:**
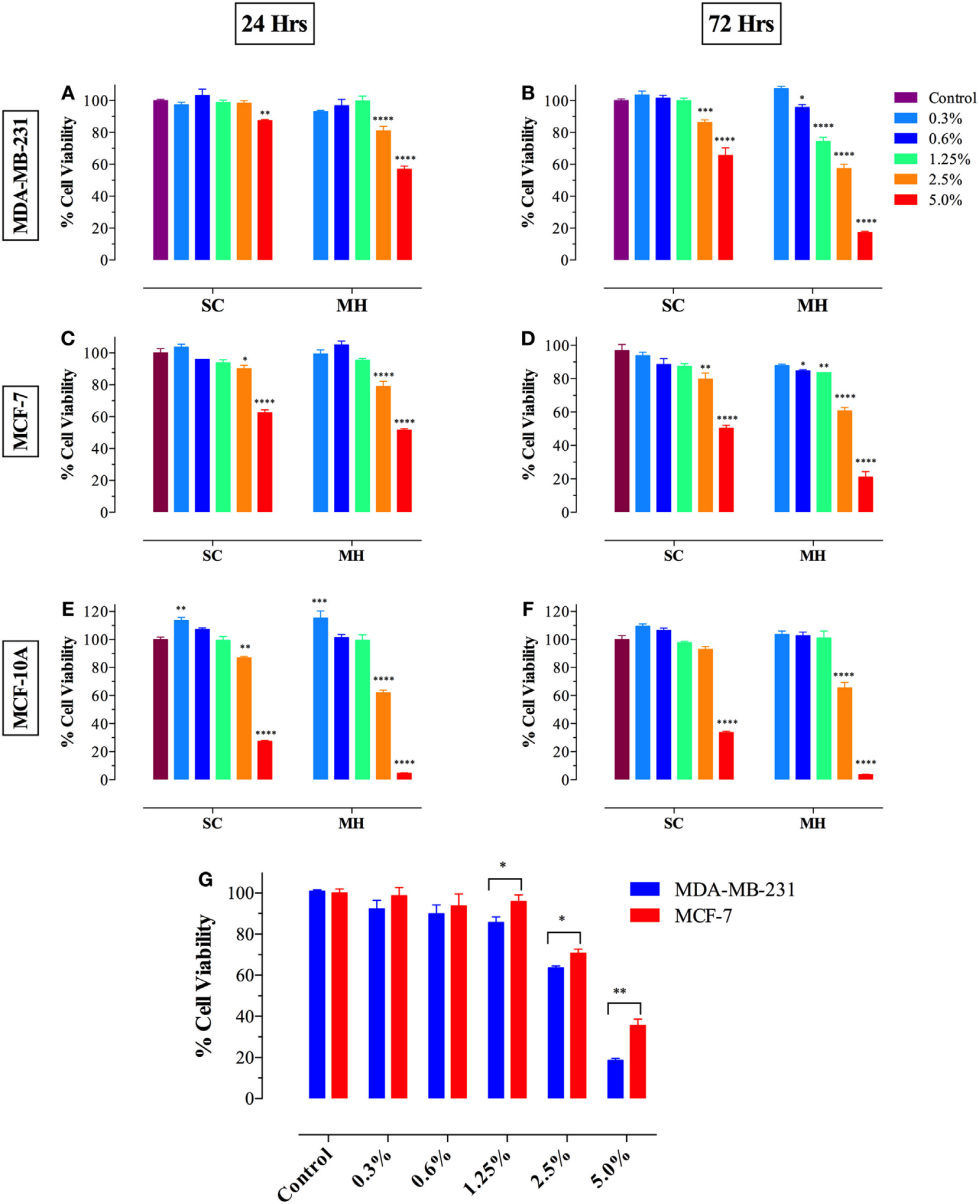
Manuka honey (MH) induces cell cytotoxicity in MDA-MB-231 [graphs **(A,B)**] and MCF-7 [graphs **(C,D)**] cancer cells but not in normal MCF-10A breast epithelial cells [graphs **(E,F)**]. MDA-MB-231 and MCF-7 cells were plated at 5 × 10^3^ cells/well and incubated for 24 [graphs **(A,C)**] or 72 h [graphs **(B,D)**] in the absence or presence of the indicated concentrations of MH (range 0.3–5.0% w/v), or equivalent sugar control (SC) solution. MCF-10A cells were plated at 2 × 10^4^ cells/well and processed similarly [graphs **(E,F)**]. **(G)** MDA-MB-231 and MCF-7 breast cancer cells were exposed to the indicted concentrations of MH or taxol (50 ng/ml) for 48 h. Cell viability was determined using CellTiter-Glo luminescent assay. Results are expressed as percentage viability (mean ± SEM) of MH or SC-treated cell cultures compared to untreated controls and are representative of three independent experiments. Asterisks denote statistically significant differences in viability of experimental groups compared to control [graphs **(A–F)**] or between the indicated experimental groups [graph **(G)**] (*p < 0.05; **p < 0.01; ***p < 0.001; ****p < 0.0001).

We first investigated the effect of exposure to different concentrations of MH (range 0.3–5.0%) on breast cancer cells following incubation for 24–72 h. As a control, we used a solution containing the three major sugars (fructose, glucose, and sucrose) at equivalent concentrations to those found in MH and designated SC solution (23). Incubation of MDA-MB-231 (Figures 1A,B) or MCF-7 (Figures 1C,D) breast cancer cells with MH resulted in significant loss of viability, which was dependent on time of exposure and concentration of MH used in culture. At 24 h, significant loss of cell viability was observed after culture with 2.5 or 5% MH in both cell lines and this was further increased after 72 h of culture. Incubation of cancer cells with equivalent concentrations of sugars (2.5 or 5% SC) also led to decreased viability, although to a lesser extent than that observed with MH. Interestingly, incubation of cancer cells with lower concentrations (0.6 and 1.25%) of MH also resulted in significantly decreased viability, which was clearly evident after 72 h of culture (Figures 1B,D). Importantly, cells incubated with equivalent SC solutions showed no significant reduction in proliferation at any time point. Moreover, we also investigated the effect of MH on the non-neoplastic MCF-10A breast epithelial cell line (Figures 1E,F). Although high concentrations (2.5 and 5%) of MH or SC were also toxic to MCF-10A cells, no significant loss of viability was observed when lower concentrations (0.6 and 1.25%) were used, even after 72 h of incubation (Figure 1F). These data demonstrate that concentrations of MH up to 1.25% were generally non-toxic to non-neoplastic cells. Furthermore, exposure of human breast cancer cells to MH resulted in a significant loss of viability that was clearly dose and time dependent. Our results suggest that MCF-7 cells were relatively more resistant to MH-induced growth inhibition compared to MDA-MB-231 cells. This is illustrated by the comparative susceptibility of both cell lines to MH at 48 h (Figure 1G). The calculated MH IC_50_ values for MDA-MB-231 cells at 48 and 72 h are 3.0 and 2.6%, respectively. The corresponding IC_50_ values for MCF-7 cells are 4.0 and 3.2%, respectively.

### Activation of Caspase-Mediated Apoptosis by MH

**Figure 2:**
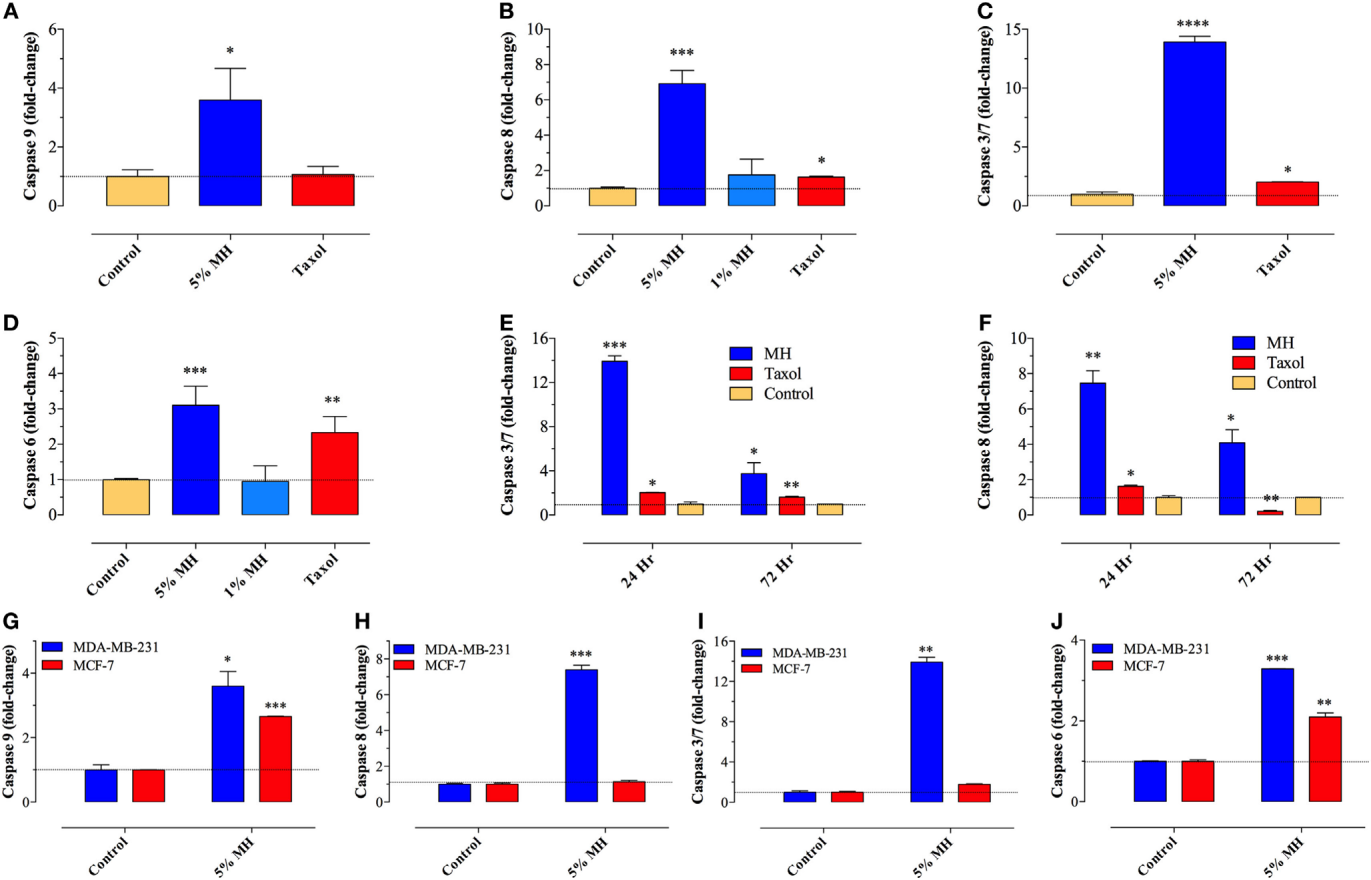
Manuka honey (MH) induces caspase-dependent apoptosis in breast cancer cells. **(A–F)** MDA-MB-231 cells were cultured in the presence or absence of MH (1 or 5% w/v) or taxol (50 ng/ml), as indicated, for 24 h. The enzymatic activity of caspase 9 [graph **(A)**], caspase 8 [graph **(B)**], caspase 3/7 [graph **(C)**], and caspase 6 [graph **(D)**] were determined using specific kits, as per manufacturer’s instructions. The effect of incubation time on enzymatic activity of caspase 3/7 [graph **(E)**] and caspase 8 [graph **(F)**] was also determined. The data are presented as mean ± SEM fold increase in caspase activity after normalization to the number of viable cells for each culture condition, and is representative of six independent experiments. **(G–J)** A comparison of the enzymatic activity of caspase 9 **(G)**, caspase 8 **(H)**, caspase 3/7 **(I)**, and caspase 6 **(J)** in MDA-MB-231 and MCF-7 cells following incubation with 5% MH for 24 h. The data are presented as mean ± SEM fold increase in caspase activity and is representative of three experiments. Asterisks denote statistically significant differences in viability of experimental groups compared to control (*p < 0.05; **p < 0.01; ***p < 0.001; ****p < 0.0001).

Previously, we demonstrated the capacity of MH to induce apoptosis in a melanoma cell line through the activation of the intrinsic pathway involving caspase 9 and caspase 3 (7). To assess the activation of caspase pathway in MDA-MB-231 and MCF-7 cells, they were incubated with up to 5% MH (the higher concentration is required to observe apoptosis over a 24 h period). Exposure of MDA-MB-231 cells to 5% MH solution resulted in the activation of both initiator caspases 9 and 8 (3.6- and 6.9-fold increase, respectively) (Figures 2A,B). As far as the executioner caspases, our findings show that incubation of MDA-MB-231 cells with MH led to the activation of caspase 3/7 and caspase 6 (13.9- and 3.1-fold increase, respectively) (Figures 2C,D), indicative of an accelerated caspase activation leading to apoptosis. The activation of initiator and executioner caspases was dependent on the concentration of MH used, as incubation of MDA-MB-231 cells with 1% solution for 24 h had a minimal effect on caspase activity (Figures 2B,D). By contrast, treatment of MDA-MB-231 cells with paclitaxel (taxol; 50 ng/ml) led to a small but significant increase in caspase 8 (1.7-fold) activity (Figures 2B,F), but no evidence for the activation of caspase 9 could be observed (Figure 2A), consistent with previous findings (7, 28). Although maximal caspase 3/7 and 8 activation was observed at 24 h, the activity of both caspases was still significantly elevated after 72 h of exposure to MH (Figures 2E,F). These findings demonstrate the capacity of MH to induce apoptosis in MDA-MB-231 cells through the activation of multiple initiator and executioner caspases. Human MCF-7 breast cancer cells have been reported to be caspase 3-deficient (29). The fact that the growth of these cells was still susceptible to inhibition by MH prompted further investigation of the exact caspase(s) involved in this cell line. The data clearly show that, unlike the case with MDA-MB-231 cells, exposure of MCF-7 cells to MH induced the activation of only caspase 9 and caspase 6 (Figures 2G–J). This suggests that MH-induced apoptosis in caspase 3-deficient cancer cells occurs through the induction of the intrinsic pathway and is mediated by the executioner caspase 6.

### Pro-Apoptotic Effect of MH on Human Breast Cancer Cells Is Independent of ROS Production

**Figure 3:**
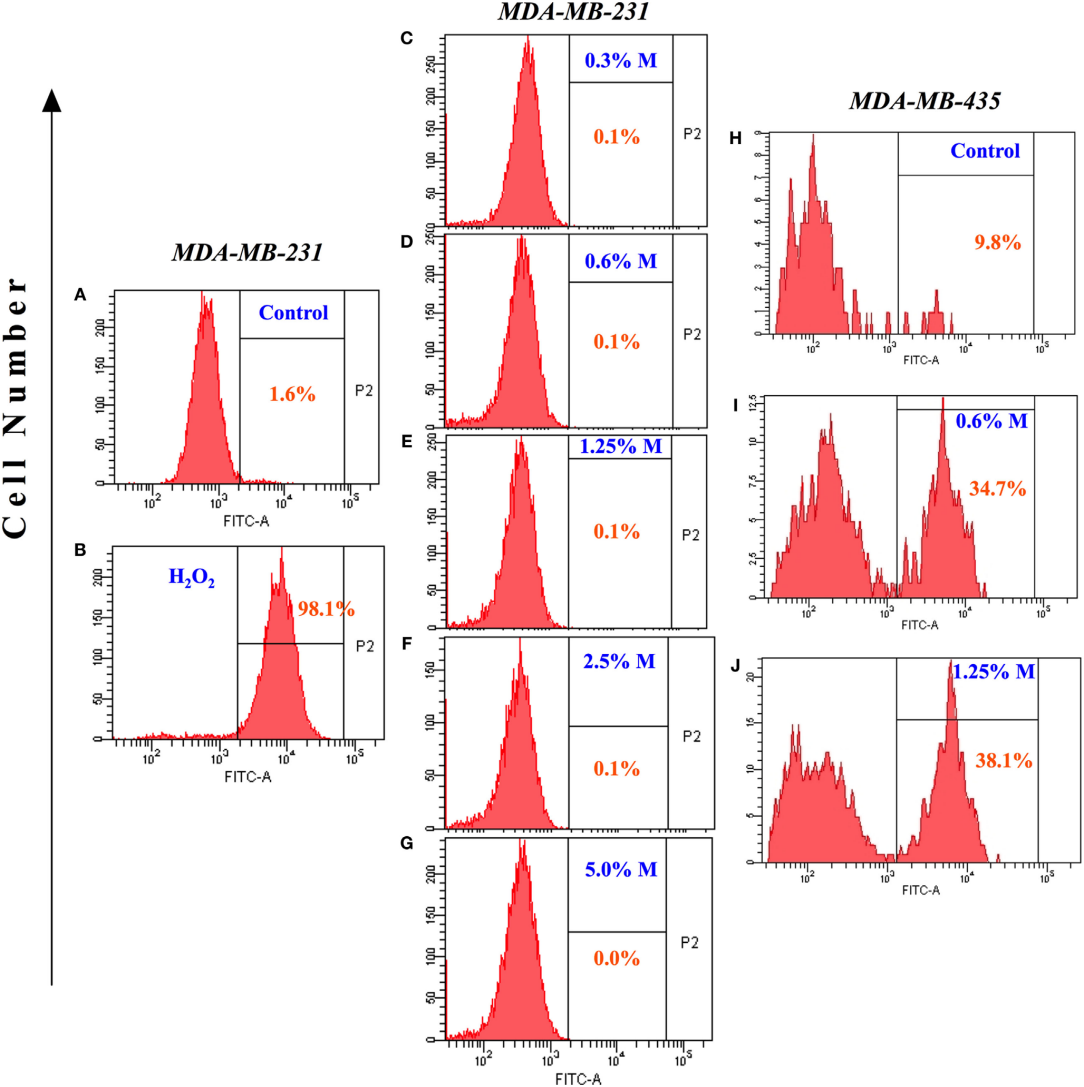
Lack of reactive oxygen species generation in manuka honey (MH)-treated breast cancer cells. MDA-MB-231 cells were incubated without **(A)** or with the indicated concentrations of MH **(C–G)** or H_2_O_2_ [positive control; **(B)**] for 24 h. Following treatment, 2′,7′-dichlorofluorescein diacetate (DCF-DA) probe was added and cells were processed for flow cytometric analysis. The percentage of positive cells in each histogram is shown by the gated population (P2 gate). **(H–J)** Histogram panels show DCF-DA staining of human MDA-MB-435 melanoma cells following treatment with MH **(I,J)** or control **(H)**. The results are representative of two independent experiments.

Increased synthesis of reactive oxygen and nitrogen radicals is indicative of a heightened state of oxidative stress and may be associated with enhanced apoptosis. We, therefore, examined whether the ability of MH to inhibit the growth of MDA-MB-231 or MCF-7 cancer cells is associated with increased production of ROS. Incubation with MH using a range of concentrations (0.3–5%) for 24 h failed to induce any intracellular ROS formation in MDA-MB-231 (Figures 3A–G) or MCF-7 (data not shown) cells. A similar analysis was carried out after 48 h of incubation with MH, but ROS production remained undetectable in both MDA-MB-231 and MCF-7 cells (data not shown). As a positive control, incubation with H_2_O_2_ led to 98% of the cells expressing ROS (Figure 3B). The lack of effect of MH on ROS production in MDA-MB-231 and MCF-7 breast cancer cells is not universal. Exposure of a human melanoma cancer cell line, MDA-MB-435, to even low concentrations (0.6–1.25%) of MH induced ROS production in 34.7 and 38.1% of the cells, respectively, in comparison to control (Figures 3H–J). Thus, MH exhibits differential ROS-inducing capacity in different cancer cells and induces apoptosis in MDA-MB-231 and MCF-7 cells independent of ROS production.

### Mechanism of MH-Induced Apoptosis of MDA-MB-231 Breast Cancer Cells

**Figure 4:**
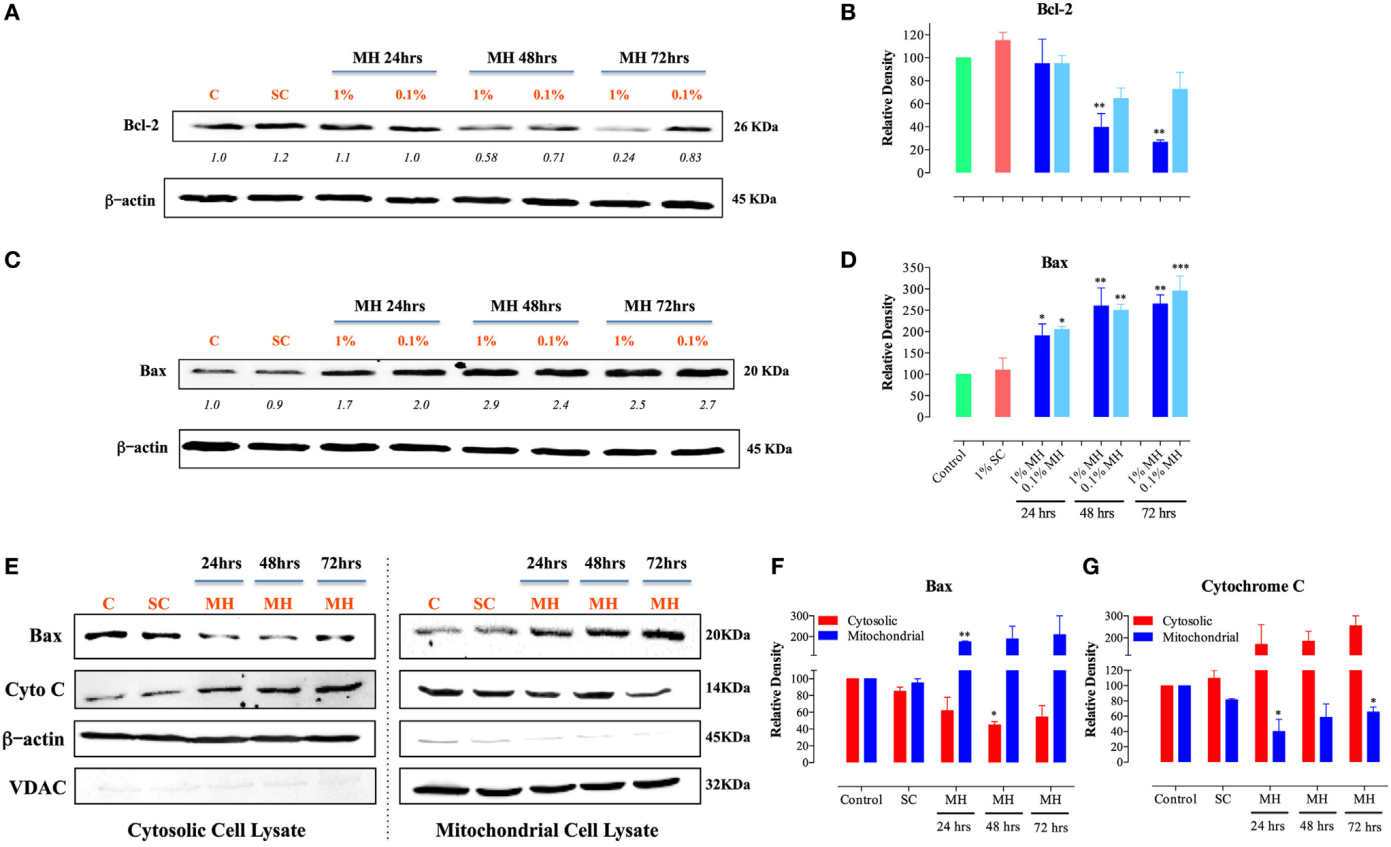
Manuka honey (MH)-induced cellular toxicity is triggered by mitochondria-associated apoptosis pathway. MDA-MB-231 cells were incubated with MH or sugar control (SC) (at 0.1 and 1% final concentrations) or cultured in medium alone for 24, 48, and 72-h. Cell extracts were then run on 12% SDS-PAGE and blots developed using antibodies specific to Bcl-2 **(A)** or Bax **(C)**. The blots were also probed with an antibody against β-actin as a control for protein loading. The numbers below the Bcl-2 and Bax blots indicate changes in band intensity, as determined by densitometric analysis. The data are representative of three independent experiments. **(B,D)** Densitometric plot analysis of Bcl-2 and Bax protein levels in MH-treated compared to control cells. The control and SC-treated cell extracts were from the 72-h-treated cell cultures (essentially identical to those from 24 to 48 h-cultures). **(E–G)** Translocation of cytochrome *c* and Bax across mitochondrial membranes following incubation of MDA-MB-231 cells with MH. Cytosolic (left panel) and mitochondrial (right panel) lysates were probed with antibodies specific to Bax and cytochrome *c*. The blots were also probed with antibodies to β-actin and VDAC as controls. The depicted data in the densitometric plots **(B,D,F,G)** represent the mean ± SEM of two to three independent experiments for each of the indicated proteins. Asterisks denote statistically significant differences between the indicated experimental group and control (*p < 0.05; **p < 0.01; ***p < 0.001).

Selective permeabilization of the mitochondrial outer membrane, which is controlled by Pro- and anti-apoptotic members of the Bcl-2 family, is a critical factor in the induction of apoptosis (30). Upon induction of apoptosis, the pro-apoptotic protein Bax translocates to mitochondrial membranes, where it inserts and mediates the release of cytochrome *c* from the intermembrane space into the cytosol, thereby triggering the activation of the caspase pathway (31, 32). Incubation of MDA-MB-231 cells with MH resulted in a gradual, and dose-dependent, decrease in the level of Bcl-2 that was first evident after 48 h of treatment (Figure 4A). The extent of the reduction in Bcl-2 level was approximately 60 and 74% after 48 or 72 h of culture with 1% MH, respectively (Figures 4A,B). However, there was no alteration in Bcl-2 levels in cells cultured with 1% SC solution for up to 72 h (Figures 4A,B). In contrast to the reduction in Bcl-2, MH induced a significant increase in the levels of Bax protein at all time points (24–72 h) tested (Figures 4C,D). We further determined the changes in Bax and cytochrome *c* levels over 24–72 h in cytosolic and mitochondrial lysates of MDA-MB-231 cells after exposure to 1% MH or SC solution. The results demonstrate that incubation with MH, but not SC, induced the translocation of Bax from the cytosol to the mitochondria which, in turn, triggers the translocation of cytochrome *c* in the opposite direction, namely from the mitochondria to the cytosol (Figures 4E–G). These findings highlight the involvement of the mitochondria in MH-induced death of breast cancer cells.

### Effect of MH on Colony Formation Capacity of MDA-MB-231 Cells

**Figure 5:**
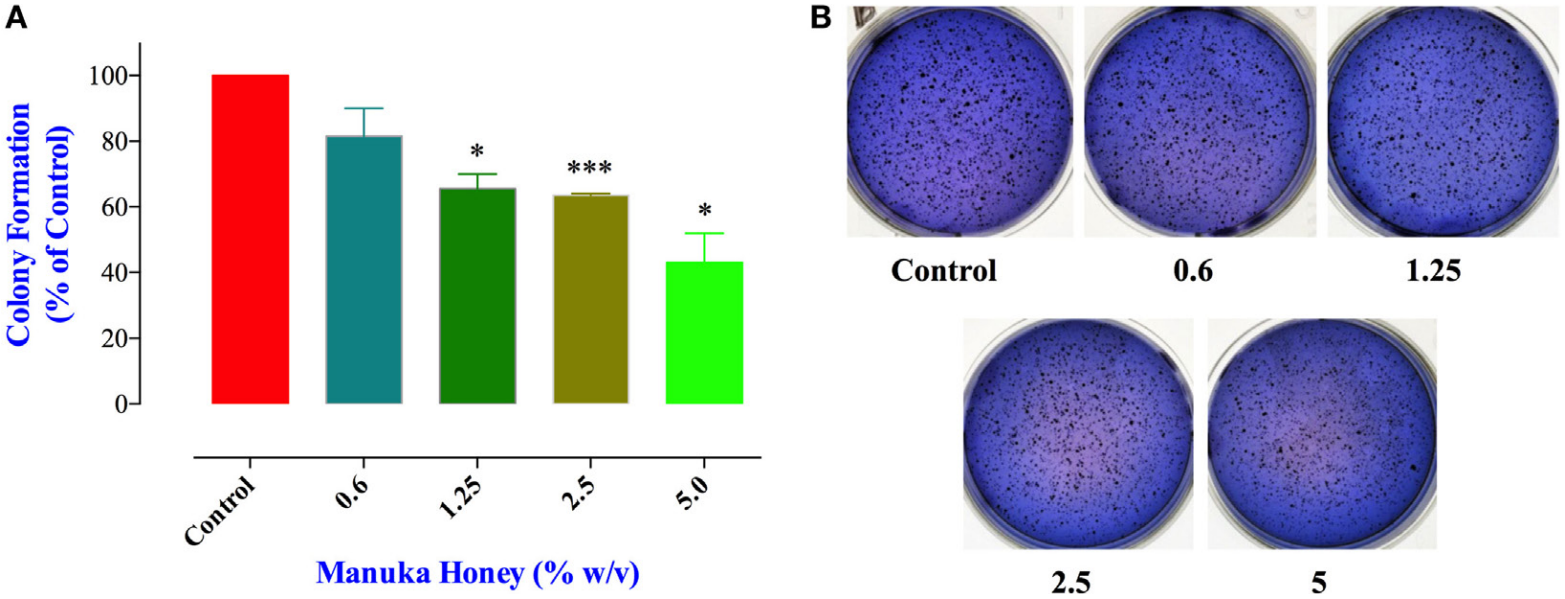
Manuka honey (MH) impairs colony growth in soft agar. **(A)** MDA-MB-231 cells were grown at a density of 5,000 cells/well in soft agar medium into 6-well plates. After 14 days, formed colonies were treated for 7 days with different concentrations of MH (0.6 and 5%). At the end, colonies were stained with Giemsa and scored as described in M&M. The data are presented as percent colonies (mean ± SEM) of MH-treated cells compared to control. **(B)** Representative pictures of the colonies formed in soft agar for each treatment group are shown. Asterisks denote statistically significant differences between the indicated experimental group and control (*p < 0.05; *** *p* < 0.001).

Cell migration and invasion together with the capacity to form colonies at distant sites are critical properties that define all metastatic tumors, such as MDA-MB-231 cancer cells. Therefore, we next addressed the effect of MH treatment on these essential functions using specific *in vitro* assays. These studies were carried out on MDA-MB-231 cells only since MCF-7 cells are known to have low migratory and invasive properties (33). The clonogenic cell survival assay determines the ability of cancer cells to proliferate indefinitely and form colonies. Treatment of MDA-MB-231 cells with MH led to a significant inhibition in colony formation in a concentration-dependent manner (Figures 5A,B). The extent of the observed inhibition was 34, 38, and 57% at MH concentrations of 1.25, 2.5, and 5%, respectively (Figure 5A). It should be noted that in this assay MH treatment had no impact on the total number of the preformed colonies (Figure 5B). Rather, exposure to MH led a significant decrease in the percentage of large colonies (>200 μm in diameter) in comparison with the control group (Figure 5A), which reflects an inhibitory effect on the colonies’ growth. These results demonstrate that MH exerted a significant inhibitory effect on long-term colony formation capacity of MDA-MB-231 cells.

### Inhibition of Cancer Cell Migration and Invasion by MH

**Figure 6:**
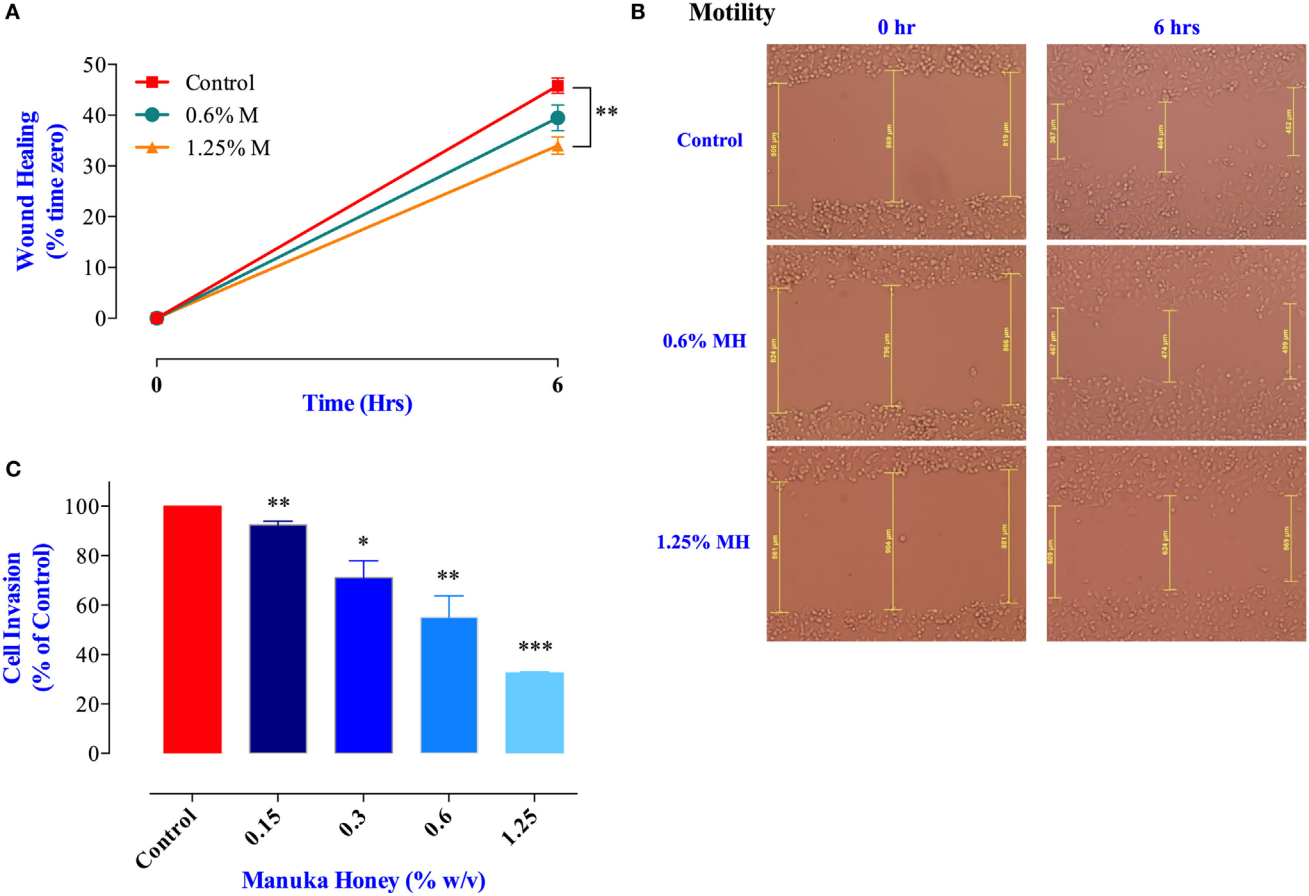
Manuka honey (MH) impairs breast cancer cell migration and invasion *in vitro*. **(A,B)** Wounds were introduced in MDA-MB-231 confluent monolayers cultured in the presence or absence (control) of MH (0.6 and 1.25%). The mean distance that cells traveled from the edge of the scraped area over a period of 6 h at 37°C was measured in a blinded fashion to determine the percentage of wound healing, as shown in panel **(B)**. The data are expressed as means ± SEM of triplicate determinations. **(C)** MDA-MB-231 cells were incubated for 24 h in the presence or absence of MH (0.15–1.25%). Cells that invaded into Matrigel were scored as described in Section “Materials and Methods.” Results are expressed as percent cell invasion (mean ± SEM) of treated cell cultures compared to control and are representative of three independent experiments. Asterisks denote statistically significant differences experimental groups compared to control (*p < 0.05; **p < 0.01; ***p < 0.001).

Cancer cell migration was examined using the wound-healing assay (34). MDA-MB-231 cells were incubated for 6 h in the presence or absence of MH (0.6 or 1.25% final concentration). These concentrations were chosen because they are known not to cause any significant apoptosis in these cells over this time period (Figure 1A). The results of the wound-healing assay show that exposure to 1.25% solution of MH led to a significant inhibition in cell migration (up to 26% within 6 h; Figures 6A,B). Exposure to 0.6% solution also led to a small (14%) but insignificant level of inhibition. Cancer cell invasion was also investigated by determining the extent of cell migration through a layer of matrigel (35). The results of this assay, shown in Figure 6C, demonstrate the level of susceptibility of this critical cancer cell function to MH exposure. MDA-MB-231 cells incubated in the presence of exceedingly small doses of MH (0.15–1.25%) were significantly inhibited in their cell invasion ability. The degree of inhibition was proportional to the concentration of MH used in the assay, with concentrations of 0.15, 0.3, 0.6, and 1.25% resulting in 9, 29, 45, and 68% inhibition, respectively (Figure 6C). These results demonstrate the heightened susceptibility of two of the critical properties of metastatic cancer cells, namely cell migration and invasion, to inhibition by exceedingly low concentrations of MH.

### Inhibition of Angiogenesis

**Figure 7:**
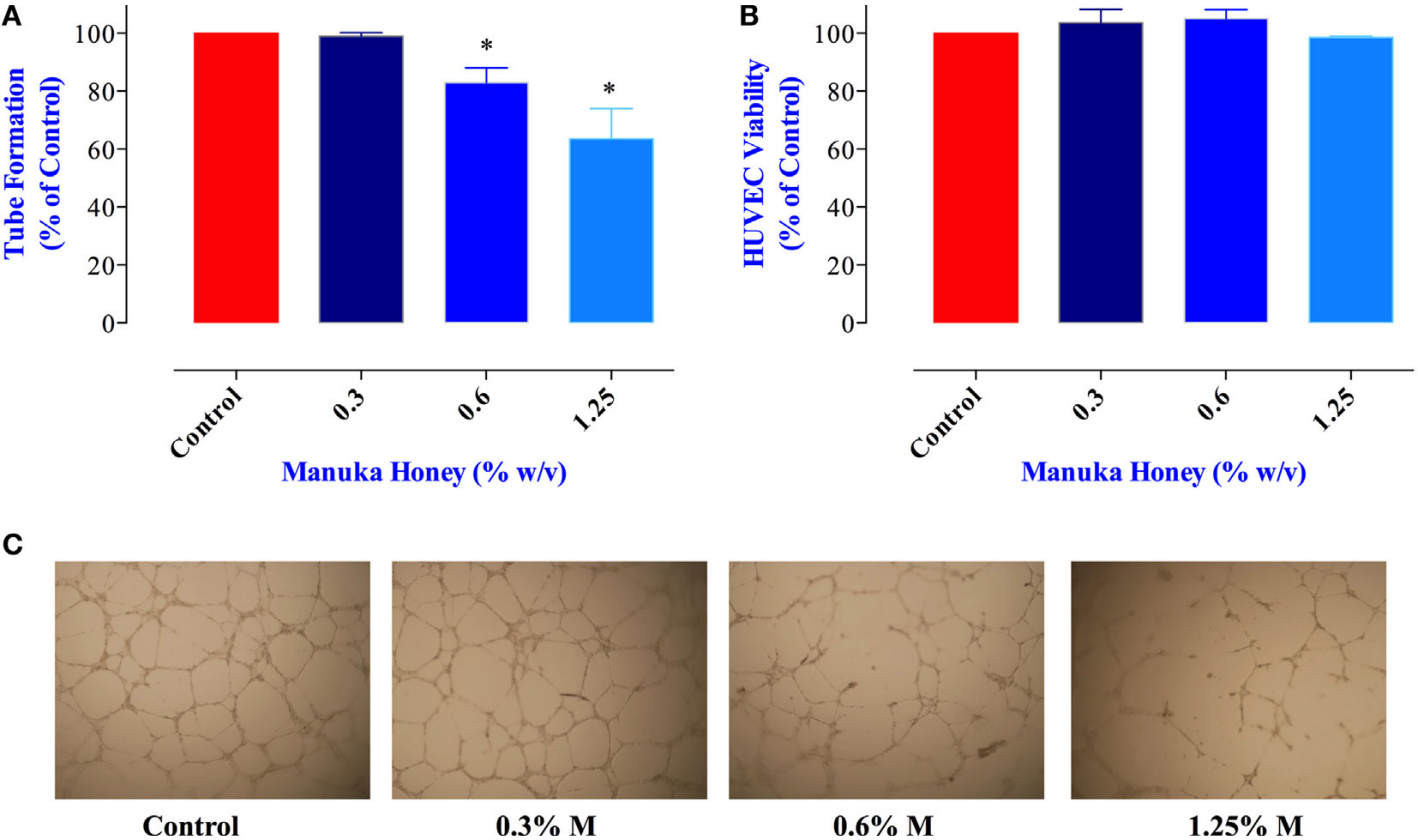
Impact of manuka honey (MH) on the formation of capillary-like structures by human umbilical vein endothelial cells (HUVECs) *in vitro*. **(A)** Quantification of tubular morphogenesis induced in HUVEC cells cultured in the absence or presence of MH. Tube formation was determined by quantifying the length of tube-like structures containing connected cells. The results are expressed as mean ± SEM of control. **(B)** HUVEC cells were treated with the indicated concentrations of MH for 8 h and viable cells assayed as described in Section “Materials and Methods.” The data are expressed as percent viable cells (mean ± SEM) in MH-treated groups compared to control. **(C)** Patterns of angiogenesis induced by HUVECs cultured on Matrigel matrix in 96-well plates in the absence or presence of MH. All experiments were repeated at least three times. Asterisks denote statistically significant differences experimental groups compared to control (*p < 0.05).

Proliferation and metastatic spread of cancer cells is dependent on the formation of an intricate network of new blood vessels within the tumor, a process known as angiogenesis (36). The potential effect of MH on tumor angiogenesis has not been explored previously. Therefore, we tested the consequence of exposure to MH on the ability of HUVECs to form vascular tubes, an *in vitro* correlate assay for angiogenesis. Incubation of HUVEC with MH at 0.6 and 1.25% final concentrations led to significant reduction (17 and 36% inhibition, respectively) in vascular tube formation (Figures 7A,C). Importantly, the observed inhibition was not due to any decrease in the viability of HUVECs (Figure 7B). Thus, we conclude that MH may also be able to influence *in vivo* tumor growth *via* a disruption of the angiogenic potential of cancer cells.

### Treatment of MDA-MB-231 and MCF-7 Cells with MH Blocks STAT3 Signaling Pathway

**Figure 8:**
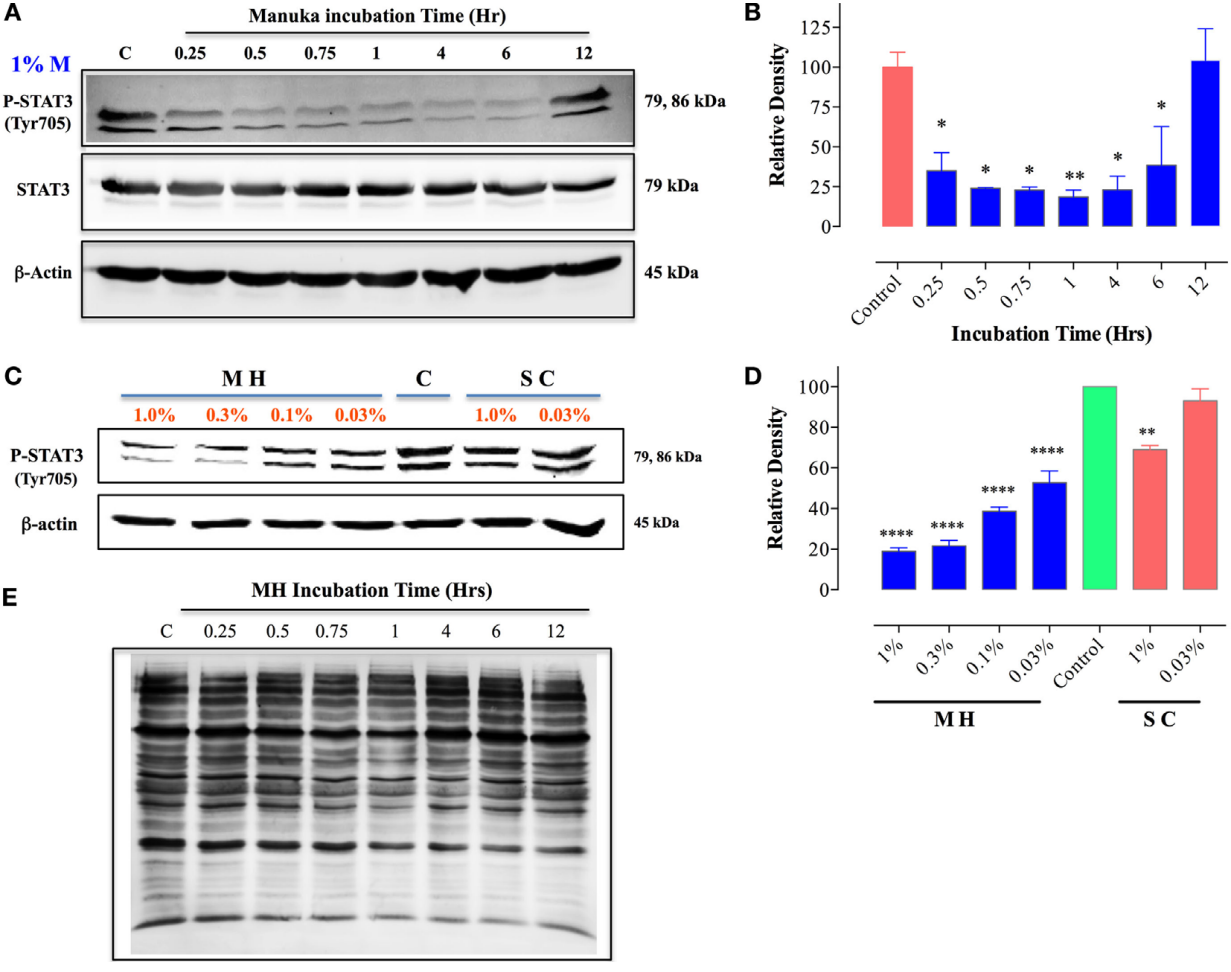
Inhibition of STAT3 activity by manuka honey (MH) treatment in MDA-MB-231 cells. Western blot analysis of total STAT3 and pY-STAT3 in MDA-MB-231 cells after exposure to 1% MH for the indicated time periods **(A)**. Whole cell extracts were resolved on 10% SDS-PAGE and immunoblotted with mAbs specific to pY-STAT3 (Tyr705) or total STAT3 protein. β-actin was used as a control for protein loading. **(B)** Densitometric analysis of band intensity of pY-STAT3 blot (Figure 8A), expressed as relative density in comparison to untreated control group. **(C)** Analysis of pY-STAT3 levels in MDA-MB-231 cells after a 1 h-incubation with different concentrations of MH or sugar control (SC) solution (range 0.03–1%) or medium alone [lane **(C)**]. **(D)** Densitometric analysis of band intensity of pY-STAT3 levels of Figure 8C in comparison to control cells. The depicted data in the graphs represent the mean ± SEM of three independent experiments. **(E)** Total tyrosine phosphorylation protein patterns in untreated and MH-treated cells. MDA-MB-231 cells were either untreated or treated with 5% MH for the indicated times. Whole cell extracts were resolved on 10% SDS-PAGE and probed with 4G10 mAb, which detects all tyrosine-phosphorylated cell proteins. The data are representative of three independent experiments. Asterisks denote statistically significant differences in protein expression of experimental groups compared to control (*p < 0.05; **p < 0.01; ****p < 0.0001).

We next investigated the underlying mechanism of how MH decreases the viability of cancer cells. Of particular interest is the identification of the earliest molecular targets of MH in breast cancer cells. MDA-MB-231 cells are known to express constitutively activated STAT3 pathway, principally through the engagement of an IL-6/IL-6R autocrine loop (37), and this pathway has been implicated in breast cancer oncogenesis (38). Therefore, we examined the consequence of MH exposure on STAT3 phosphorylation. As early as 15 min after incubation with 1% (w/v) MH solution, the level of active pY-STAT3 was reduced by 65% compared to the level observed in untreated cells, and by 1 h the level of inhibition was >80% (Figures 8A,B). The inhibition of pY-STAT3 persisted for at least 6 h and returned to normal levels by 12 h, presumably due to continuous cell growth and utilization of the inhibitory factors in MH. Importantly, MH treatment had no significant effect on total STAT3 protein levels at any of the time points examined (Figure 8A). Next, we determined the sensitivity of pY-STAT3 inhibition following 1 h-exposure to differing concentrations (range 1–0.03%) of MH or SC solution (Figure 8C). Significant inhibition of pY-STAT3 was evident following culture with MH at all doses, with the pY-STAT3 levels ranging from 19.0 ± 3.0 to 52.7 ± 10.1% (mean ± SEM) of control following exposure to 1 or 0.03% MH concentration, respectively (Figures 8C,D). Importantly, incubation of MDA-MB-231 cells with SC solution resulted in only a marginal reduction (69.0 ± 2.8% of control) in pY-STAT3 levels which was observed only at the highest concentration (1%) of SC used (Figures 8C,D). These findings demonstrate that incubation of MDA-MB-231 cells with MH at concentrations as low as 0.03% results in a significant and specific reduction in pY-STAT3 levels. Tyrosine phosphorylation of cytosolic proteins is a widely utilized mechanism for the regulation of cell cycle and signal transduction pathways in eukaryotic cells (39, 40). Therefore, we wished to examine the global pattern of tyrosine-phosphorylated proteins in cancer cells following treatment with MH. The results of this analysis revealed no evidence that the total protein tyrosine phosphorylation pattern is grossly altered in MDA-MB-231 cells exposed to 1 (data not shown) or 5% MH (Figure 8E), suggesting that the targeted effect on pY-STAT3 is specific.

**Figure 9:**
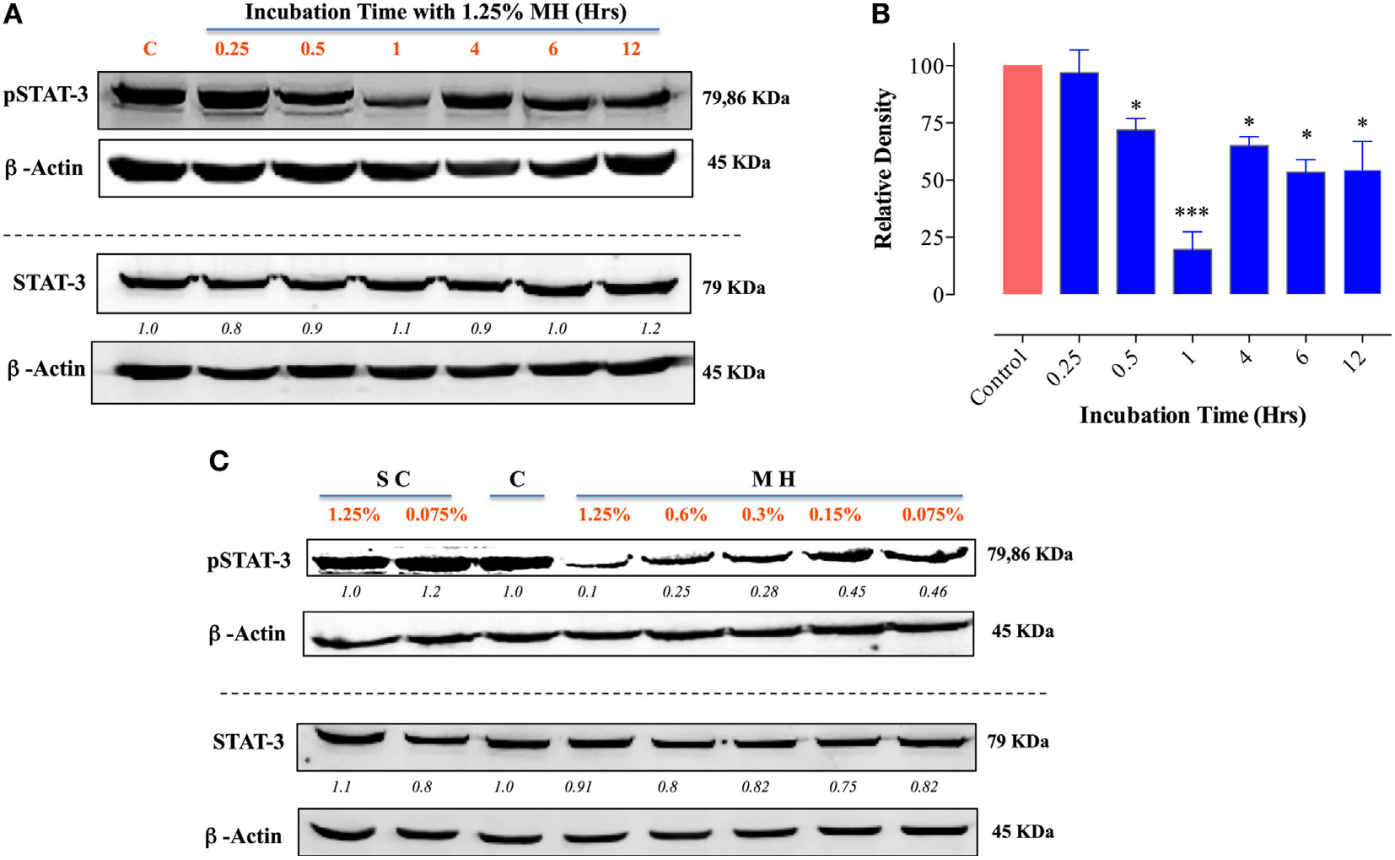
Exposure to manuka honey (MH) inhibits pY-STAT3 activity in MCF-7 cells. Western blot analysis of total STAT3 and pY-STAT3 was carried out as in Figure 8 using MCF-7 cells after exposure to 1.25% MH for the indicated time periods **(A)**. Whole cell extracts were resolved on 10% SDS-PAGE and immunoblotted with mAbs specific to pY-STAT3 (Tyr705) or total STAT3 protein. β-actin was used as a control for protein loading. The data are representative of two independent experiments. **(B)** Densitometric analysis of band intensity of pY-STAT3. The depicted data are based on two independent blots. **(C)** Analysis of pY-STAT3 and total STAT3 levels in MCF-7 cells after a 1 h-incubation with different concentrations of MH or sugar control (SC) solution (range 0.075–1.25%) or medium alone [lane **(C)**]. Asterisks denote statistically significant differences in protein expression of experimental groups compared to control (*p < 0.05; ***p < 0.001).

MCF-7 cells are known to express low levels of constitutive pY-STAT3 (41, 42). Nevertheless, a similar analysis of the effect of MH on pY-STAT3 in MCF-7 cells revealed very similar findings to those in MDA-MB-231 cells (Figure 9). Within 30 min of exposure to 1.25% MH, the level of pY-STAT3 was reduced by ~30% compared to the level observed in untreated cells, with maximal inhibition (80.3%) occurring at 1 h of incubation (Figures 9A,B). Despite a partial recovery in pY-STAT3 levels by 4 h, they were still significantly inhibited (55% of normal level) even after 12 h of treatment (Figures 9A,B). Similar to the results in MDA-MB-231 cells, MH treatment had no significant effect on total STAT3 protein levels within this time frame (Figure 9A). The sensitivity of pY-STAT3 inhibition following a 1 h-exposure to differing concentrations (range 1.25–0.075%) of MH or SC solution (Figure 9C) was also examined. The levels of pY-STAT3, but not total STAT3, were dramatically reduced following exposure to MH in a dose-dependent manner (Figure 9C). The degree of inhibition ranged from 90% at 1.25% MH to 54% at the lowest MH concentration of 0.075% (Figure 9C). Moreover, incubation of MCF-7 cells with SC solution had no effect on pY-STAT3 levels at the same concentration range (Figure 9C).

### Inhibition of IL-6 Production and Secretion in Breast Cancer Cells

**Figure 10:**
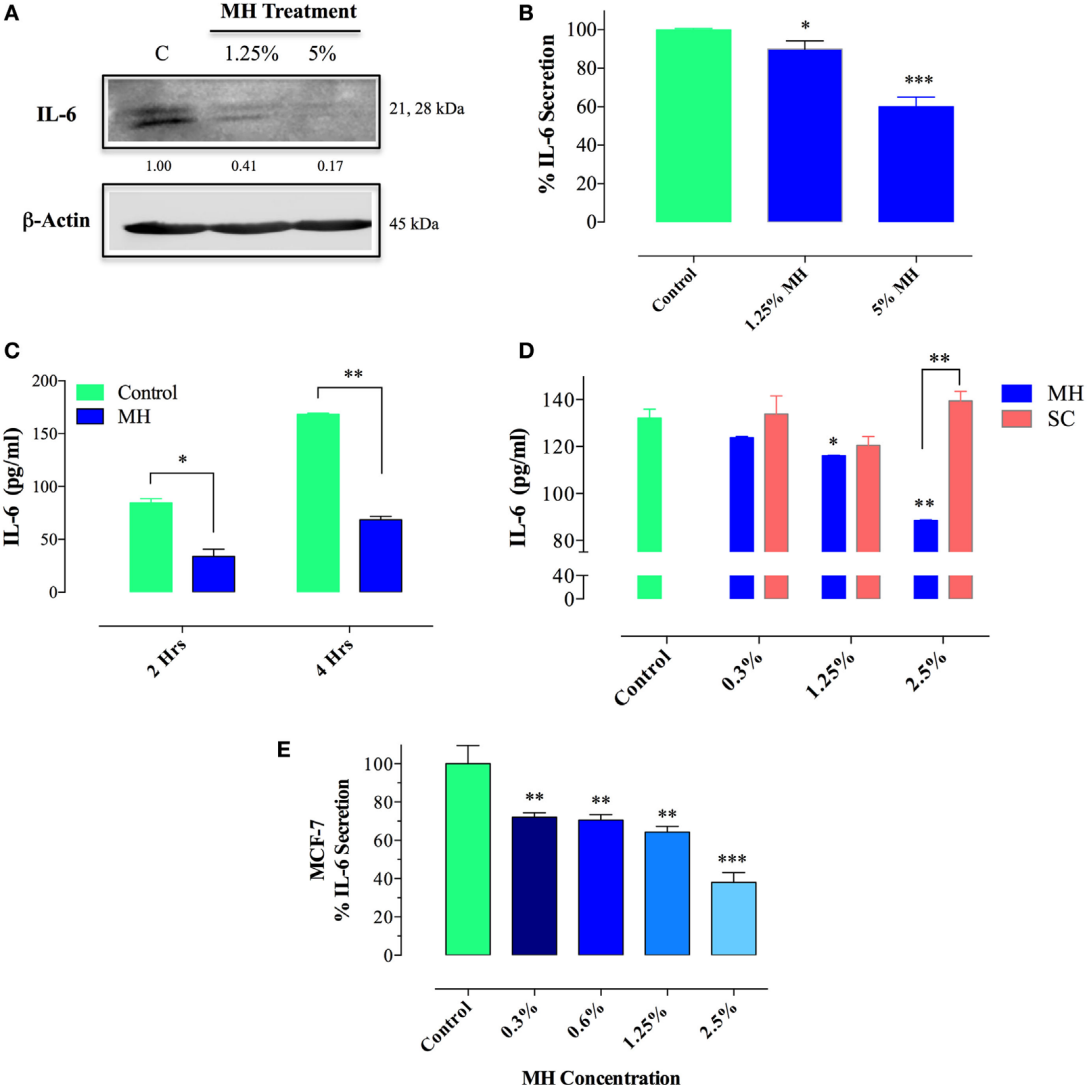
Treatment with manuka honey (MH), but not sugar control (SC) solution, inhibits interleukin-6 (IL-6) secretion and production in breast cancer cells. **(A)** Western blot analysis of IL-6 synthesis. MDA-MB-231 cells were incubated in the presence or absence of MH (1.25 or 5%) for 4.5 h, with Brefeldin A added for the last 4 h of culture. Extracts were run on 10% SDS-PAGE and immunoblotted with a mAb specific to IL-6. The numbers below the blot indicate changes in band intensity, as determined by densitometric analysis. The data are representative of two independent experiments. **(B–E)** Analysis of IL-6 secretion in MDA-MB-231 **(B–D)** and MCF-7 **(E)** cells by ELISA. MDA-MB-231 cells were cultured for 12 h with or without the indicated concentrations of MH **(B)** or exposed to 5% MH for 2 or 4 h **(C)**. In a separate experiment, cells were cultured with indicated concentrations of MH or SC solution for 4 h **(D)**. Similarly, MCF-7 cells were cultured with the indicated MH concentrations for 4 h **(E)**. After culture, cell-free supernatants were collected and analyzed for IL-6 content by ELISA. The data are representative of four (MDA-MB-231 cells) or two (MCF-7 cells) independent experiments. Asterisks denote statistically significant differences in IL-6 levels of experimental groups compared to control (*p < 0.05; **p < 0.01; ***p < 0.001).

Since IL-6/IL-6R signaling is thought to be primarily responsible for the constitutive pY-STAT3 in MDA-MB-231 cells, we next sought to determine the effect of MH on IL-6 synthesis. Cells were incubated with MH (at 1.25 or 5% final concentration) for 4.5 h in the presence of Brefeldin A, an inhibitor of protein egress from the endoplasmic reticulum. The presence of IL-6 in total cell extracts was then determined by Western blots. The data, illustrated in Figure 10A, demonstrate that treatment with MH inhibits IL-6 synthesis in a dose-dependent manner. Relative to untreated control cells, the amount of detectable IL-6 protein was reduced by 59 and 83% in cells incubated with 1.25 or 5% MH solution, respectively (Figure 10A). Inhibition of IL-6 production was also observed at the level of IL-6 protein in culture supernatants, as determined by ELISA. The addition of MH to MDA-MB-231 cells resulted in a significant, dose-dependent, inhibition of IL-6 secretion after overnight incubation (Figure 10B). Moreover, significant suppression of IL-6 secretion (~60%) was observed as early as 2–4 h following cell culture in the presence of MH (Figure 10C), demonstrating the rapid kinetics of this inhibition. Furthermore, we investigated the potential effect of SC solution on IL-6 secretion. Unlike MH, incubation of MDA-MB-231 cells with up to 2.5% SC solution had no significant effect on IL-6 secretion (Figure 10D). Finally, we analyzed the effect of MH treatment on IL-6 production in MCF-7 cells. Similar to MDA-MB-231 cells, culture of MCF-7 cells in the presence of MH resulted in dose-dependent inhibition of IL-6 secretion, ranging from 28% inhibition at 0.3% MH to 62% at 2.5% MH (Figure 10E). We conclude that exposure of MDA-MB-231 and MCF-7 breast cancer cells to MH leads to inhibition of IL-6 production as well as secretion. This inhibition is quite independent of the sugar content of MH.

## Discussion

Honey possesses antioxidant, anti-inflammatory, anti-microbial, and anti-tumor properties that have led to an increased interest in studying and characterizing their underlying mechanisms (7, 8, 43, 44). Previously, we reported that MH has anti-proliferative capacity against a number of human and murine cancer cell lines that was attributed to its ability to induce caspase-mediated apoptosis (7). The characteristics that define successful cancerous tumors include not only the capacity for continuous, self-sufficient proliferation but also increased resistance to apoptosis, acquisition of migration and invasion capabilities, and pro-angiogenic potential (45). Human TNBC represent a major challenge in treatment owing to their inherent resistance to chemotherapy and high capacity for metastatic spread (46, 47). The objective of this study was to investigate the influence of MH on the growth of two distinct human breast cancer cell lines, the TNBC MDA-MB-231 cells and ER-positive MCF-7 cells, and characterize the earliest molecular targets involved. In addition, the effect of MH on other critical functions of human TNBC, such as migration and invasion capacity, and pro-angiogenic potential, was investigated in metastatic MDA-MB-231 cells. In addition to its anti-proliferative activity on both MDA-MB-231 and MCF-7 cells, we demonstrate the capacity of MH to inhibit colony formation, migration, and invasion of MDA-MB-231 cells. Exposure to MH induced mitochondria-mediated cell death. This was evidenced by the translocation of Bax into the mitochondria, the release of cytochrome *c* into the cytosol and the activation of initiator caspases 8 and 9 as well as the executioner caspases 6 and 3, leading to accelerated apoptosis. Interestingly, honey-induced apoptosis of MDA-MB-231 and MCF-7 mammary cancer cells appears to be independent of ROS generation, which is unlike the case with human melanoma and colon cancer [this study; (14)]. Furthermore, our findings demonstrate that a short-term exposure (as little as 15 min) to MH caused a reduction in pY-STAT3 and blocked subsequent IL-6 synthesis in both MDA-MB-231 and MCF-7 cells, thereby identifying the IL-6/STAT3 autocrine growth pathway as a potential main target of MH in human breast cancer cells. The fact that MH affected the same pathway in the two different cell lines suggests that its action is independent of hormone receptor expression in breast cancer cells.

Interleukin-6 is a proinflammatory cytokine with pleiotropic functions in regulating not only different aspects of immune responses but also the growth and differentiation of different types of cancer cells, including breast, liver, and colon cancers (48, 49). The binding of IL-6 to its receptor, IL-6Rα, induces the recruitment of the signal transducing receptor gp130 to the complex and initiates the activation of Janus kinases (JAK1 and JAK2). This, in turn, catalyzes the tyrosine phosphorylation of the transcription factor STAT3 (signal transducer and activator of transcription), which dimerizes and translocates to the nucleus thereby initiating a complex transcriptional set that acts to promote cell growth and inhibit apoptosis (50). High levels of IL-6 are expressed in malignant breast cancers where they, together with breast stromal fibroblasts, drive both autocrine and paracrine growth through IL-6/IL-6R/STAT3 positive feedback loop (51–53). Moreover, growth of MDA-MB-231 TNBC cells is regulated through the coordinate autocrine production of IL-6 and IL-8 (54). IL-6 has also been implicated in the malignant transformation of breast cancer stem cells as well as in the enhancement of cancer cell metastatic potential and epithelial to mesenchymal transition (55, 56). Constitutively activated STAT3 has been described in many human breast cancer cell lines and in 40–50% of primary human breast tumors (38, 41), making it an attractive target for the development of potential anti-cancer therapies. Using microarray gene expression analysis, we have preliminary evidence demonstrating highly significant inhibition of a number of genes involved in the IL-6/STAT-3 pathway (such as IL-6ST, IL-6R, and SOCS3) in MH-treated human breast cancer cells (al-Ramadi et al., unpublished observations). These preliminary data confirm and extend the findings reported in this study.

Signaling through STAT3 increases the expression of many genes involved in cancer cell proliferation, survival, migration, invasion, and angiogenesis. For example, the STAT3 transcription factor induces the expression of matrix metalloproteinase 2 (MMP-2), MMP-9, and epithelial–mesenchymal transition-related genes in promoting cancer cell invasion and metastasis (57). In addition to IL-6, the STAT3 pathway is utilized by another closely related member of the IL-6 cytokine family, namely IL-11. This cytokine utilizes STAT3 as a transcriptional regulator of many genes that confer tumor-defining characteristics in breast cancer, including survival, proliferation, invasion, angiogenesis, and metastasis (58). Elevated levels of IL-11 have been documented in primary human breast cancer as well as in breast cancer cell lines and are associated with poor prognosis in breast cancer patients (58). Expression of IL-11 endows breast cancer cells with the capacity to metastasize, particularly to the bone (59). Several factors are known to regulate the expression of IL-11 in breast cancer, including Ras oncogene and tumor hypoxia (60, 61). However, inhibition of STAT3 abrogates Ras-induced IL-11 transcription in breast cancer (60). Thus, given the central role of the IL-6/IL-11–STAT3 pathway in the regulation of breast cancer progression and metastasis, blocking of this pathway by MH may underlie the latter’s multi-faceted effects that are reported in this study. Nevertheless, it is also important to note that the observed inhibition in cancer cell migration and invasion as well as angiogenesis occurred at concentrations of MH that were not cytotoxic, suggesting perhaps a delinking between the anti-proliferative capacity of MH on breast cancer cells and its effects on other pro-tumoral functions.

Different types of honey have been reported to have anti-proliferative activities against breast cancer cells, principally through the induction of caspase-mediated apoptosis (8). The multiple activities of MH on cancer cells are most likely due to its flavonoids and phenolic acid constituents. Recently, a comprehensive quantitative analysis of 31 different New Zealand MH samples revealed that 61% of the total flavonoid content is accounted for by just four major flavonoids, namely pinobanskin, pinocembrin, luteolin, and chrysin (62). In that study, other flavonoid compounds, such as quercetin, kaempferol, and galangin, were also detected but at 10- to 20-fold lower levels. Anti-tumor activities have been ascribed to many of the phenolic constituents in honey, including chrysin (13), luteolin (15), and quercetin (63, 64). In terms of underlying mechanisms, chrysin has been shown to inhibit the metastatic and invasive potential of TNBC, while quercetin acts as a phytoestrogen to induce apoptosis in hormone receptor-positive breast cancer cells (63). Interestingly, luteolin has been found to exert its anti-tumor effects by targeting and inactivating STAT3 in breast (65), liver (66), and pancreatic cancers (67). Luteolin binds to HSP90 and suppresses STAT3 phosphorylation that, in turn, leads to increased Fas/CD95 expression and caspase 8-dependent apoptosis (65, 66). Taken together, the different effector capacities of these flavonoid compounds may explain our current findings showing the effectiveness of MH in suppressing multiple important functions of breast cancer cells.

It has been suggested that combinations of flavonoids yield synergistic and pleiotropic effects against cancer cells that exceed the sum of the effects of the individual compounds (68). Since several groups reported on the role of luteolin in inhibiting STAT3 phosphorylation (65–67), we wished to compare the effective doses of luteolin that led to STAT3 inhibition in the different studies with the estimated dose of luteolin in MH used in this study. As mentioned earlier, luteolin is one of only four major flavonoids found in MH that, together, account for 61% of total flavonoid content (62). The effective dose of luteolin used in the different studies was 50 µM, which approximates to a final concentration of 14.3 µg/ml. The reported mean concentration of luteolin derived from the analysis of 31 different New Zealand MH was 0.136 mg/100 g honey (62). Accordingly, the estimated concentration of luteolin in the 1% solution of MH that was used in our study is approximately 0.0136 µg/ml, which is more than 1,000-fold lower than when used individually. The fact that we have observed substantial reduction in pY-STAT3 levels even at MH concentrations as low as 0.03% demonstrates the superior nature of pY-STAT3 inhibition by MH. Notwithstanding this rather simplistic comparison, and acknowledging that MH may have more than one active flavonoid component targeting STAT3, this nevertheless confirms that inactivation of STAT3 by treatment with MH is much more effective than with the use of individual flavonoid compounds.

**Figure 11:**
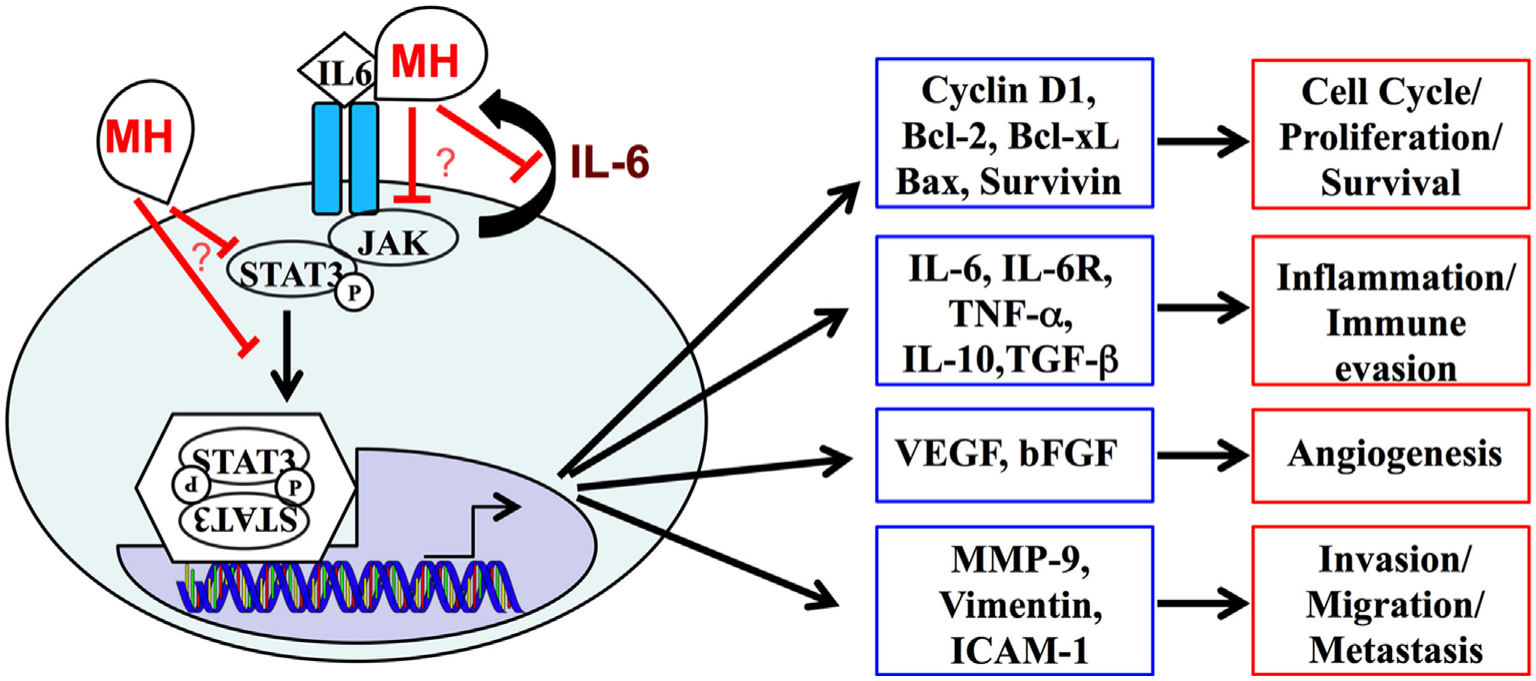
A schematic summary of the interleukin-6 (IL-6)/STAT3 signaling pathway, highlighting its central role in regulating the expression of multiple genes and cellular functions in metastatic breast cancer cells. Our data demonstrate the capacity of manuka honey (MH) to inhibit pY-STAT3, thereby suppressing this signaling pathway. The mechanistic details of how MH reduces cytoplasmic pY-STAT3 levels remain to be determined.

In conclusion, this study demonstrates that multiple effector functions of human TNBC cells can be readily inhibited by treatment with MH and identifies the IL-6/STAT3 signaling pathway as a key target of MH in these cells (Figure 11). The potential use of MH, or its bioactive constituents, to improve treatment for TNBC merits further evaluation.

## Author Contributions

PA, SA-Q, JG, KA, KR, YM, MA-D, and AA-S performed the experiments. PA, SA, and KR analyzed the data. MF-C supervised the experimental work, analyzed data, and wrote the manuscript. Ba-R designed the study, supervised the project, analyzed data, and wrote the manuscript. All authors reviewed the manuscript.

## Conflict of Interest Statement

The authors declare that the research was conducted in the absence of any commercial or financial relationships that could be construed as a potential conflict of interest.

## Abbreviations

MH, manuka honey; TNBC, triple-negative breast cancer; ER, estrogen receptor; ROS, reactive oxygen species; STAT3, signal transducer and activator of transcription 3; pY-STAT3, phosphorylated STAT3; HUVECs, human umbilical vein endothelial cells; SC, sugar control.
